# Editorial: Sociologies of health and emotions

**DOI:** 10.3389/fsoc.2024.1388509

**Published:** 2024-03-05

**Authors:** Marci Cottingham, Rebecca E. Olson, Gillian Bendelow

**Affiliations:** ^1^Department of Sociology, Kenyon College, Gambier, OH, United States; ^2^The University of Queensland, Brisbane, QLD, Australia; ^3^University of Brighton, Brighton, United Kingdom

**Keywords:** sociology, health, carework, emotion, emotional labor

Illness experiences are imbued with emotions for those receiving care, those giving care, and those bearing witness. Emotions, as the crucial link between mind and body (Hochschild, 1983), are implicated in the social divisions (gender, class, and race) that characterize work within the healthcare professions. In the wake of the COVID-19 pandemic, ongoing economic constraints, the geopolitical sequelae of climate change and war, healthcare work has seemingly never been more emotionally demanding. With a rise in mental health concerns, the interplay of emotions, social conditions and interactions demand renewed attention. This Frontiers Research Topic explores these intersections across the *Sociologies of health and emotions*: how emotions and emotional discourses shape social relations and health outcomes.

Across all papers within the Research Topic, one unifying theme relates to the drawbacks—and even dangers—of conceptualizing emotions as individualistic phenomena within health/care. Brown et al. engage with this theme through challenging the widespread view in psychiatry of hope as a positive individual resource. Taking a more ambiguous and interactionist appreciation of hope, they examine how it features in interviews with service-users and professionals from three psychosis services in England. They phenomenologically position hope as a tool co-constructed by clinicians and service-users for bracketing fears in the present, helping an individual to endure conditions in the present. Hope, they argue, becomes problematic—for clinicians and service-users—when inflated, leaving service-users open to excess vulnerability and undermining clinicians' longer-term coping.

Rodriquez and Harvey and Stacey—in differing settings—both attend to the drawbacks of considering clinical empathy as an individual skill. Rodriquez examines the dangers of viewing emotions as merely interactional strategies. Drawing on interviews with staff in ICUs overwhelmed with COVID-19 patients, Rodriquez explicates how the uncertainty, intensity and futility of care for COVID-19 patients prompted moral distress, emotional exhaustion and burnout amongst staff. In particular, Rodriquez argues that clinical empathy should not be viewed as a performance, but as central facet of health care work, highlighting the perils of prioritizing economic efficiencies above staff wellbeing. Ultimately, Rodriquez calls for research that draws on an understanding of empathy as an emotional practice (Cottingham, 2022). Harvey and Stacey respond to that call.

Drawing on Cottingham's (2022) “emotion practice” framework to view emotions as resources that can be fostered, but also depleted, Harvey and Stacey attend to patient care and clinician wellbeing as layered and interconnected acts. In analyzing interviews with medical students and their instructors at one US-based university, they identify the incongruency in the skills-based approach to empathy training in medical education in a compassion-impoverished curriculum. Both medical students and educators positioned medical school as a difficult environment for fostering (skills in) empathy, arguing that “it is difficult to cultivate kindness in an unkind place” (p. 8). The potential insincerity cultivated could risk exacerbating the mental health crisis described by Rodriquez, with burnout and poor mental health linked to inauthenticity and surface acting (Hochschild, 1983).

Dillon et al. and Mescouto et al. attend to the Research Topic's overarching theme by drawing on relational conceptualizations of emotion (Ahmed, 2014) to reframe low-back pain and mental health care in Australia as more than individualistic or dyadic: as circulating, political, cultural and racialized affective assemblages. In Dillon et al.'s critical reflexive ethnographic examination, they trace the embodied assemblages of care and distress. Findings frame distress as an everyday experience, rather than a pathology. Dillon et al. show that it is not just patients that experience distress in clinical interactions, but physiotherapists too, with patients and clinicians affecting each other. Such findings foreground the importance of how emotions are conceptualized to how care is framed and delivered. Mescouto et al.'s findings draw on emotion to reveal how care is conceptualized and who deserves such care. Their work attends to emotions such as hate, anger and indifference in interviews with staff and clients connected to providing non-clinical psychosocial support to culturally and linguistically diverse people experiencing mental ill-health. Such affective intensities underscore the conservative sociopolitical logics underpinning care. “Hate” for any doubling up of service provision for refugee clients, for example, exposes presumptions about who (class, race, citizenship status) should receive how much support.

Byrne et al. and Francis and Ghafurian both make use of new technological developments in their research on emotions and healthcare. Byrne et al. investigate the experiences and emotion management of hospital doctors in Ireland during COVID-19. They combine the old with the new, merging long-established theorizing on alienation and depersonalization with new communication technologies [Mobile Instant Messaging Ethnography (MIME)] that allow researchers to better connect with participants. They find that doctors use strategies of acquiescence and depersonalization in order to cope with the range of negative emotions (guilt, pressure, anger, resentment) they feel. Francis and Ghafurian develop an innovative software application that allows dementia caseworkers to benefit from insights from Affect Control Theory (ACT). Based on the claims of ACT, they pull out salient identities and relevant behaviors based on dementia patients' biographies in order to better guide healthcare workers in relationally interacting with patients.

While Brown et al., Dillon et al., and Mescouto et al. include service users and patients in their analysis, Wechuli goes a step further in centring the experiences of disabled people. Wechuli integrates the sociology of emotions with disability studies to better understand how medicalization discourses influence the experiences of the disabled and chronically ill. Wechuli critically examines the various harms that medicalization discourses perpetuate, including harm through dismissal of legitimate feelings of discrimination, love, and desire. Far from individualistic phenomena, in this conceptual paper, we can see how intertwined emotional assumptions are with health knowledge and practices.

Overall, in this Frontiers Research Topic we showcase theory-driven papers which foreground complex conceptualisations of emotion, and their multi-layered role in the underpinning of relations that define health care experiences and its delivery in contemporary societies.

## Author contributions

MC: Writing—original draft, Writing—review & editing. RO: Writing—original draft, Writing—review & editing. GB: Writing—original draft, Writing—review & editing.
